# Comparing and Optimizing RNA Extraction from the Pancreas of Diabetic and Healthy Rats for Gene Expression Analyses

**DOI:** 10.3390/genes13050881

**Published:** 2022-05-14

**Authors:** Amani M. Al-Adsani, Sahar A. Barhoush, Nasmah K. Bastaki, Suzanne A. Al-Bustan, Khaled K. Al-Qattan

**Affiliations:** Department of Biological Sciences, Faculty of Science, Kuwait University, Kuwait City 13060, Kuwait; sahar.barhoush@ku.edu.kw (S.A.B.); nasmah.bastaki@ku.edu.kw (N.K.B.); s.albustan@ku.edu.kw (S.A.A.-B.); khaled.alqattan@ku.edu.kw (K.K.A.-Q.)

**Keywords:** pancreas, RNA extraction, RNA isolation, RIN value, STZ-diabetic rat model

## Abstract

Advanced differential gene expression analysis requires high-quality RNA. However, isolating intact pancreatic RNA is challenging due to abundant pancreatic ribonucleases, which limits efficient downstream gene expression analysis. RNA*later* treatment reduces endogenous ribonucleases effects through either pre-organ excision via organ mass or bile duct direct injection or organ mass injection post-isolation. We compared RNA extraction protocols to establish a reproducible and effective pancreatic RNA extraction method to obtain high RNA integrity number (RIN) values from healthy and streptozotocin (STZ)-induced diabetic rats for gene expression analyses. Different methods were tested focusing on RNase activity inhibition using RNA*later* (Qiagen) pre-harvest of the pancreatic tissue, and extracted RNA quality and concentration were analyzed using NanoDrop spectrophotometer, Agilent Bioanalyzer, and RT-PCR. Inclusion of several pre- and post-excision modifications in the RNeasy Mini Kit (Qiagen) protocol resulted in RIN values more than two-fold higher compared to those using the standard protocol. Additionally, RT-PCR amplification of the housekeeping gene, *β-actin*, revealed no differences in extracted RNA quality from healthy and STZ-induced diabetic rats. We compared and developed a more effective and reproducible pancreatic RNA extraction method from healthy and diabetic rats, which resulted in RNA of superior quality and integrity and is suitable for complex molecular investigations.

## 1. Introduction

Advanced differential gene expression analysis requires high-quality RNA. Although RNA extraction from various tissues and cells is a straightforward process, isolating RNA with a high RNA integrity number (RIN) from the pancreas is challenging, mainly because the pancreas is a dual-functioning gland comprising an endocrine and exocrine acinar portion that is rich in digestive enzymes, including ribonuclease (RNase) [1]. During the dissection of the pancreas, acinar cell granules are disrupted and release their contents, causing tissue autolysis and affecting the yield and quality of RNA. During an assessment of total RNase activity in tissues from different organs in rodents, the pancreas showed a 181,000-fold increase in activity as compared to that in the liver, which in turn had a 64-fold increase relative to that in the brain (Krosting, J. and Latham, G. (2005). RNase Activity in Mouse Tissue: Classification, Hierarchy, and Methods for Control. Ambion TechNotes, 12 (3)).

Isolation methods for pancreatic RNA are inconsistent in their reproducibility and unstandardized, although several studies have reported the use of commercially available extraction kits based on guanidinium thiocyanate-phenol-chloroform in combination with silica column-based solid extraction methods [2] (Table 1). In this study, we aimed to establish a reproducible and effective method for extracting RNA with high RIN values from the pancreas of healthy and streptozotocin (STZ)-induced diabetic rats, which is suitable for microarray analyses. Several modifications in RNA extraction methods reported in previous studies were combined to devise a single protocol for pre- and post-excision of rat pancreatic tissue samples using a commercially available kit. These modifications resulted in RNA with consistent and improved RIN values suitable for downstream RNA analysis.

## 2. Materials and Methods

### 2.1. Animal Care and Handling

Healthy male Sprague-Dawley rats (Harlan Laboratories, Derby, UK) were of about 6–7 weeks of age (weighing approximately 160–180 g each) at the start of the experiment. The rats were housed in the Animal Care Facility under regular ambient conditions (22–24 °C, 30–35% humidity and natural light/dark cycle~12:12 h) before and during the study and handled according to the Instructions Guide for the Care and Use of Laboratory Animals [9]. Type 1 diabetes mellitus was chemically induced in six rats by intraperitoneal injection of a single dose of STZ solution (Sigma, St. Louis, MO, USA) (60 mg/kg in 0.5 mL of 0.01 M citrate buffer, pH 4.5) after a 2-h fasting period.

### 2.2. Rat Sacrifice and Pancreatic Tissue Collection

Following anesthesia (0.2 mL/100 g) with a mixture of ketamine (9 mL, 10%, Dutch farm Nedar, Host den Berg, Holland) and xylazine (1 mL, 10%, Interchemie, Vernary, Holland), 18 normal rats (NR) and six diabetic rats (DR) were prepped for pancreas pre-treatment and collection.

### 2.3. RNAlater Infusion

**Figure 1:**
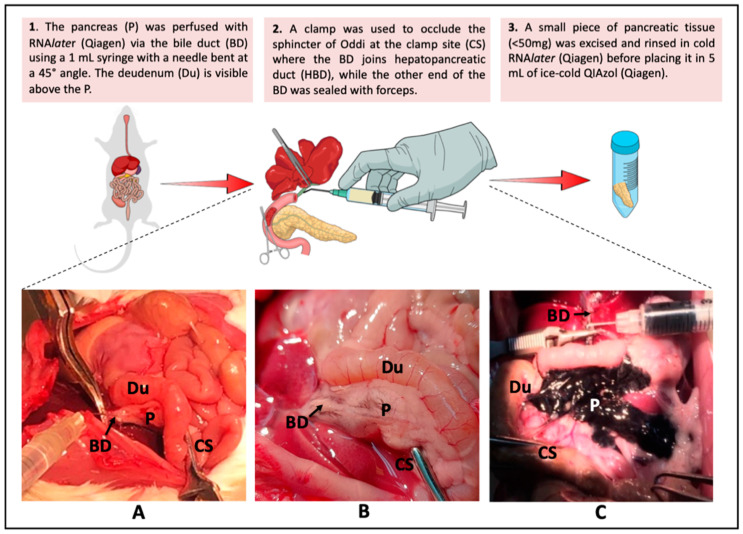
RNA*later* (Qiagen) injection into the bile duct (BD). (**A**) The liver was flipped over and pushed aside to expose the pancreas (P). (**B**) The BD was located and traced distally to its point of joining with the pancreatic duct (hepatopancreatic duct: HPD) at the sphincter of Oddi and occluded with a clamp at the clamp site (CS). RNA*later* (Qiagen) was injected by inserting a diabetic syringe needle into the BD. (**C**) To confirm the effectiveness of the RNA*later* (Qiagen) perfusion technique, Indian ink was injected into a test pancreas, showing good perfusion. The diagrams in the figure were generated using mindthegraph.com. Accessed on 10 December 2021.

Each anesthetized rat was secured on its dorsal side and a thorough sterilization of its ventral side using 70% alcohol was performed before exposing the abdominal viscera by a longitudinal ventral incision. The liver was flipped over and pushed aside; the bile duct (BD, Franklin Lake, NJ, USA) was located and traced to its joining point with the pancreatic duct (hepatopancreatic duct [HPD], Figure 1A). Further, a 1-mL syringe (BD, Franklin Lake, NJ, USA) with a 30-G needle (BD, Franklin Lake, NJ, USA) (bent to 45° angle) was inserted into the lumen of the BD and the pancreas infused with RNA*later* (Qiagen, Hilden, Germany) (Figure 1A). To ensure complete perfusion, the HPD was occluded with a clamp just above the region where it joins the duodenum (sphincter of Oddi). In addition, forceps were used to hold and seal the other end of the BD to prevent retrograde perfusion of the liver (Figure 1A,B). The location of the RNA*later* (Qiagen) infusion site and clamping of the sphincter of Oddi were performed as previously described [4] and resulted in good perfusion, as indicated by the swelling of the pancreas (Figure 1B). Prior to administering RNA*later* (Qiagen), the perfusion technique was tested by infusing India ink (Loba Chemie, Colaba, India), which showed a very good distribution in the pancreas (Figure 1C).

### 2.4. Isolation of the Pancreas

All dissection and surgical tools were sterilized and washed with RNaseZAP (Sigma-Aldrich, St. Louis, MO, USA) prior to use for tissue collection. Extreme caution was taken when detaching the pancreas from the attached tissues to prevent rupture and subsequent release of ribonucleases. A small piece of tissue (<50 mg) was excised from the perfused pancreas and immediately placed in 5 mL of ice-cold QIAzol (Qiagen, Hilden, Germany) contained in a sterile 50-mL centrifuge tube immersed in ice.

### 2.5. RNA Extraction

**Figure 2:**
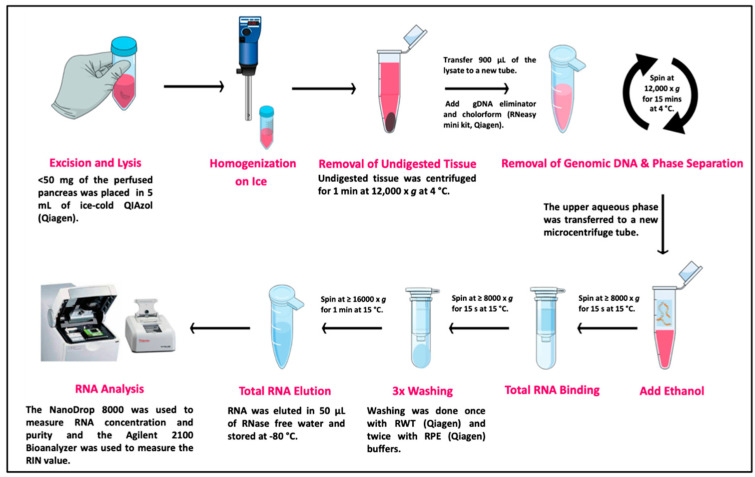
The optimized RNA extraction protocol workflow. The diagrams in the figure were generated using mindthegraph.com, accessed on 21 April 2022. RIN, RNA integrity number.

Initially, pancreatic RNA was extracted using the TriPure isolation reagent (Roche, Basel, Switzerland). RNeasy Mini Kit (Qiagen, Hilden, Germany) was also used following the manufacturer’s instructions and with modifications to improve RNA quality. The tissue was rapidly homogenized using a TissueRuptor Ultra-Turrax T8 (IKA laboratories, Staufen, Germany) for 30–60 s on ice, pausing for a few seconds every 20 s to avoid heating, until the lysate was uniformly homogeneous. An additional centrifugation step was performed for 1 min at 12,000× *g* at 4 °C to remove any unhomogenized lysate, as previously described [8]. This step replaced another step in the standard protocol in which the homogenate was incubated at room temperature (between 21–22 °C) for 5 min. The quick removal of undigested tissue at low temperature is critical to reduce any possible source of RNase contamination that could cause RNA degradation. The upper transparent aqueous layer was removed using a pipette and transferred to a sterilized microcentrifuge tube following the manufacturer’s instructions for genomic DNA elimination. This was followed by addition of chloroform to the samples, which were then centrifuged for 15 min at 12,000× *g* at 4 °C to separate the upper aqueous phase (~500 µL) containing the RNA. Then, the aqueous phase was transferred to a sterile microcentrifuge tube, and an equal volume of 70% ethanol was added to it prior to being transferred to an RNeasy spin column. The addition of 70% ethanol provides ideal binding conditions for the silica membrane of the spin column. Following two centrifugations (≥8000× *g* at 15 °C) for 15 s to remove the ethanol, the column was washed once with 700 µL RWT washing buffer (Qiagen) and twice with 500 µL RPE washing buffer (Qiagen). During each wash, the spin column was centrifuged at ≥8000× *g* at 15 °C, and the flow-through was discarded. Afterward, the column was centrifuged at 16,000× *g* for 1 min at 15 °C to eliminate any buffer or ethanol carryover. Total RNA was eluted in RNase-free water and stored at −80 °C until future use. All steps and modifications are summarized in Figure 2.

### 2.6. Analyses of Extracted RNA

RNA concentration (ng/µL) and purity at ratios A260/A280 and A260/A230 were recorded using a NanoDrop 8000 spectrophotometer (Thermo Fisher Scientific, Waltham, MA, USA). RIN values were determined using an Agilent *RNA 6000* Nano Kit using an Agilent 2100 Bioanalyzer (Agilent Technologies, Santa Clara, CA, USA).

### 2.7. Reverse Transcription and cDNA Quality Assessment

Thirteen RNA samples from pancreatic tissue extracted using the standard and optimized RNA protocols, and one control RNA sample extracted from liver tissue using the standard protocol, were used for reverse transcription (RT)-polymerase chain reaction (PCR). The SuperScript^®^ IV First-Strand Synthesis System for RT-PCR (Invitrogen, Carlsbad, CA, USA) was used to convert RNA to cDNA by mixing 300 ng/µL template RNA with Oligo d(T)_20_ primer, dNTP, and RNase-free water. RNA-primer solutions were heated at 65 °C for 5 min in an automated thermocycler (Applied Biosystems Fast Thermal Cycler Version 1.01, Life Technologies), followed by incubation on ice for 1 min. This was followed by addition of the RT reaction mixture to the annealed RNA and incubation at 23 °C followed by 50–55 °C for 10 min each, and finally at 80 °C for 10 min to inactivate the reaction. Beta-actin (*β-actin*) gene was amplified in all samples in a reaction volume of 25 µL that included 1× PCR buffer (20 mM Tris, 50 mM KCl), 3 mM MgCl_2_, 0.5 mM dNTP mix, 0.3 µM each of forward XAHR 17: 5′ CGGAACCGCTCATTGCC 3′ and reverse XAHR 20: 5′ ACCCACACTGTGCCCATCTA 3′ *β-actin* primers, 50 mU/µL *Taq* DNA polymerase, 17.5 µL H_2_O, and 0.5 µL RT template. The PCR thermal profile was as follows: initial denaturation at 94 °C for 5 min, followed by 42 cycles of denaturation at 94 °C for 30 s, annealing at 55 °C for 30 s, extension at 72 °C for 1 min, followed by a final extension at 72 °C for 7 min. The quality of the *β-actin* amplicon (289 bp) in all 13 samples was visualized and assessed on a 1% agarose gel prepared in 1× Tris/Borate/EDTA (TBE) buffer stained with 3 µL of 10 mg/mL ethidium bromide (Promega, Madison, WI, USA).

### 2.8. Statistical Analysis

The integrity and quality of RNA measurements are presented as mean ± standard error of the mean (SEM). The data of the different groups, samples, and protocols were compared using one-way analysis of variance (ANOVA) for RNA analysis followed by Fisher’s LSD test using GraphPad Prism version 9.2.0.332. Differences among readings were considered significant at *p* < 0.05.

## 3. Results

### 3.1. Evaluating the Integrity of RNA from Pancreata of Healthy Rats Using Different Extraction Protocols

Two methods were tested for pancreatic RNA extraction: the TriPure isolation reagent (Roche) and the RNeasy Mini Kit (Qiagen). Both methods yielded similar RIN values of 3.7 and 3.6, respectively. However, unlike the RNAeasy Mini Kit (Qiagen), the TriPure isolation reagent (Roche) repeatedly produced variable results and low-quality and degraded RNA (A260/A230 of 1.00 ± 0.13). Although RNA extracted using the RNeasy Mini Kit (Qiagen) resulted in improved quality and purity, the RIN value remained low at 3.6. Therefore, it is not recommended for downstream applications, such as microarray analysis, where the RIN should ideally be >8. Therefore, to increase the RIN value, protocols reported in previous studies were tested to optimize RNA extraction, along with some modifications in the RNA extraction protocol of the RNeasy Mini Kit (Qiagen).

**Figure 3:**
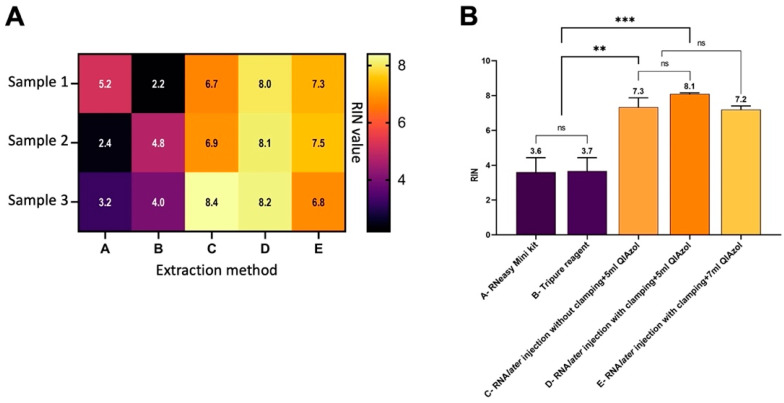
Comparison of RNA integrity number (RIN) values of pancreatic RNA samples from healthy rats following different excision techniques and extraction protocols demonstrated as (**A**) a heat map with the three RIN values of each protocol reported inside boxes and (**B**) a bar graph showing the significant difference in RNA integrity between the optimized and standard protocols using the standard one-way analysis of variance (ANOVA) for RNA analysis followed by Fisher’s LSD test. ns, non-significant (*p* > 0.05) or significant at ** *p* < 0.01, *** *p* < 0.001.

**Figure 4:**
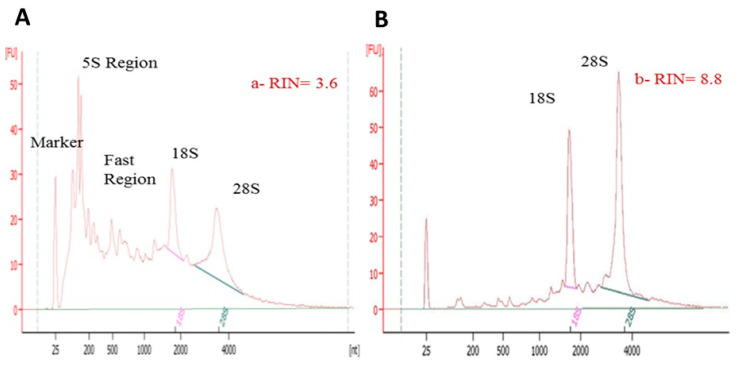
Electropherograms generated by an Agilent 2100 Bioanalyzer using the pico kit showing (**A**) RNA extracted using a standard protocol with an RIN = 3.6 and high peaks in the 5S and fast region, which indicates RNase degradation as compared with (**B**) RNA extracted using our optimized protocol that had low peaks in the same regions with two clear 18S and 28S regions, indicating intact RNA with RIN = 8.8.

**Figure 5:**
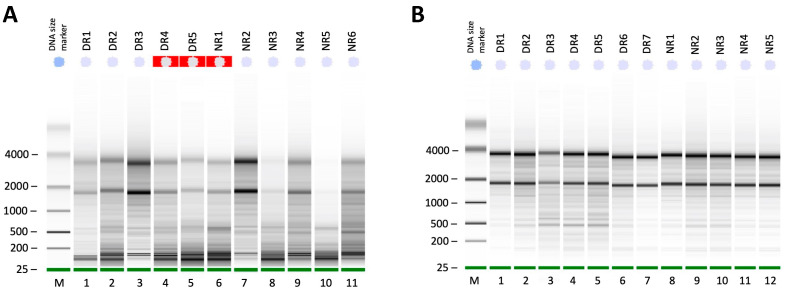
Virtual gel-like images generated by an Agilent 2100 Bioanalyzer using the pico and nano kits. (**A**) There was a high level of RNA degradation in samples extracted using the standard RNeasy mini protocol (Qiagen). Samples isolated from diabetic rats (DR) are presented in lanes 1–3, which had an RIN range from 4.2–7, while those from normal rats (NR) in lanes 7–11 had an RIN value between 2.3–7.3. Red highlighted lanes 4–6 show samples with low RIN values that could not be calculated due to degradation or sample contamination. (**B**) Intact RNA with very low levels of degradation in samples extracted using our optimized method. Samples isolated from diabetic rats (DR) are presented in lanes 1–7, which had an RIN value range from 6.7–8.8, while those from normal rats (NR) in lanes 8–12 had an RIN value ≥8.0 except for NR2 and NR3 (RIN = 7.4, 7.2), which was due to a technical difficulty during the homogenization step.

Our optimized RNA extraction method resulted in a significant increase in pancreatic RIN values (Table 2 and Figure 3). Perfusion of the rat pancreas with RNA*later* (Qiagen) significantly increased the RIN values (*p <* 0.01) in protocol C (7.3 ± 0.5) as compared to that in the standard protocol A (3.6 ± 0.8) (Table 2). Additionally, clamping the HPD and securing the BD with forceps (Figure 1) prior to RNA*later* (Qiagen) perfusion also significantly increased the RIN values in optimized protocol D (8.1 ± 0.1) as compared to that in the standard protocol (*p* < 0.001) (Table 2 and Figure 3B). Furthermore, increasing the volume of QIAzol from 5 mL in protocol D (RIN 8.1 ± 0.1) to 7 mL in protocol E resulted in lower and fluctuating RIN values with a mean of 7.2 ± 0.2 (Table 2 and Figure 3A). All RNA samples (*n* = 12) extracted using the optimized method had strong 18S and 28S peaks (Figure 4) as well as clear, sharp bands with minimal RNA degradation as compared to RNA samples extracted using the standard protocol when measured using the Agilent 2100 Bioanalyzer (Figure 5).

### 3.2. Comparison of RNA Yields between Healthy and Diabetic Rats

**Figure 6:**
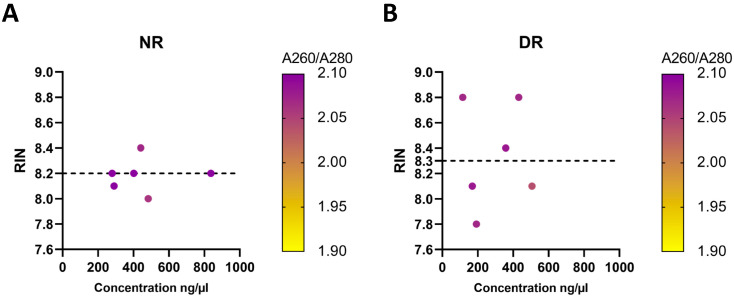
The quantity (ng/µL), quality (absorbance ratio A260/A280 nm), and integrity (RIN) of samples extracted from (**A**) normal rats (NR) (*n* = 6) and (**B**) diabetic rats (DR) (*n* = 6) using our optimized protocol. The dashed horizontal line represents the mean RIN.

To validate and confirm whether the RNA extracted using the optimized method would be suitable for gene expression analysis and generate reliable data, RNA was extracted from the pancreas of healthy (*n* = 6) and diabetic rats (*n* = 6) using our optimized method. However, differences in RNA concentrations were observed between the two groups: RNA extracted from healthy rats had a higher concentration (455.0 ± 83.2 ng/µL) than that extracted from diabetic rats (295.1 ± 64.8 ng/µL) (Table 3). Moreover, the RIN values of pancreatic samples from diabetic rats were on average slightly higher (8.3 ± 0.17) than those (8.2 ± 0.05) from healthy rats (Table 3 and Figure 6). These results indicated that the optimized method is reliable even in the diabetic model.

### 3.3. Evaluation of Different RNA Extraction Protocols Using RT-PCR

**Figure 7:**
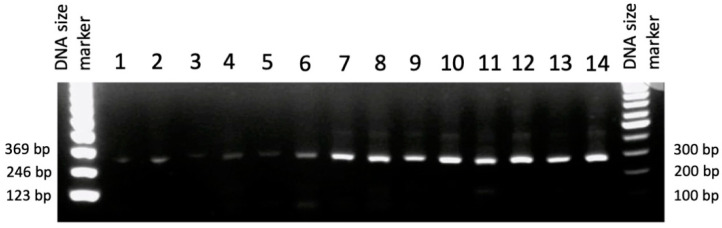
Agarose gel electrophoresis of *β-actin* amplicons (289 bp) produced from cDNA that was synthesized from RNA extracted from rat pancreas isolates. RNA was extracted from the pancreas of normal rats using the standard RNeasy Mini Kit protocol (Qiagen) (lanes 1–3); RNA*later* (Qiagen) injection without clamping and treatment with 5 mL QIAzol (Qiagen) (lanes 4 and 5); clamping and RNA*later* (Qiagen) injection followed by the optimized protocol with 7 mL QIAzol (Qiagen) (lanes 6 and 7). As a positive control, RNA was extracted from rat liver using the standard protocol (lane 8). *β-actin* was also amplified from cDNA that was synthesized from RNA isolated from pancreas of diabetic rats using clamping and RNA*later* (Qiagen) injection followed by the optimized protocols with 5 mL QIAzol (Qiagen) (lanes 9–11), or from that of normal rat pancreas samples after clamping and RNA*later* (Qiagen) injection using the optimized protocol with 5 mL QIAzol (Qiagen) (lanes 12–14). A 100-bp DNA size marker (Agilent Technologies, Santa Clara, CA, USA) and a 123-bp DNA ladder (left) were used as references.

The integrity of RNA extracted using different protocols was compared by assessing the quality of RT-PCR products of the housekeeping gene, *β-actin*. A significant increase in the band intensity of *β-actin* was observed in pancreatic RNA samples extracted from healthy rats using our optimized protocol (Figure 7, lanes 12–14) as compared to those extracted using the standard protocol (Figure 7, lanes 1–3) or the optimized protocol without clamping (Figure 7, lanes 4–5), which showed faint and unclear bands. In addition, the quality of amplicons of cDNA synthesized from RNA extracted using 7 mL QIAzol varied between samples (Figure 7, lanes 6–7) echoing the inconsistent RIN values observed (Figure 3A, column E). When we compared the integrity of RNA extracted from healthy and diseased rats, no differences were detected between samples from diabetic (Figure 6, lanes 9–11) and non-diabetic rats (Figure 7, lanes 12–14). These results suggest that our modified extraction protocol produced the best *β-actin* amplification, thus reflecting the high quality of RNA isolated from healthy and STZ-induced diabetic rats.

## 4. Discussion

Extracting RNA from pancreatic tissue is a challenging procedure because of its high RNase content that causes RNA degradation as soon as the pancreas is dissected out. Several studies have tested various pancreatic RNA extraction protocols proposing several modifications produced inconsistent results [4,5] or resulted in low RIN values [6,8]. To obtain high-quality RNA (RIN > 8) suitable for advanced downstream gene expression analysis, we tested two RNA extraction protocols that use the standard phenol/guanidine thiocyanate lysis reagent: the commercially available RNeasy Mini Kit (Qiagen) and TriPure isolation reagent (Roche). However, the quality of the pancreatic RNA obtained using either of the two protocols was not high enough (RIN approximately 3.7) (Table 2, methods A and B; Figure 3, column A and B). Extraction using TriPure isolation reagent (Roche) resulted in RNA of low quality probably because of organic compound contamination (Table 2, method B; Figure 3, column B). To improve the quality of the extracted RNA, we used the RNeasy Mini Kit (Qiagen) along with some modifications that had been reported to be effective in previously described studies [4,8]. The improved protocol does not require the preparation of more reagents but includes additional steps in the pre- and post-RNA extraction phases of the manufacturer’s protocol.

In a 2006 study, Mullin et al. [4] developed a protocol for mouse pancreatic RNA extraction using TRIzol but with inconsistent results and low purity of the extracted RNA (260/280 ratio = 1.48). Nevertheless, their method reported certain modifications in the procedure that solved problems associated with pancreatic RNA extraction. The first modification involved injecting the pancreas with RNA*later* prior to tissue isolation, while the second is a post-excision step and involved increasing the TRIzol volume from 1 to 5 mL. Briefly, their pre-excision step included in situ ductal perfusion of RNA*later* while clamping the BD at the sphincter of Oddi through which it enters the duodenum. This procedure was included in our protocol (Figure 1) and resulted in almost doubling the RIN values from 3.7 to 7.3. Clamping during perfusion of the pancreas also contributed to increased RIN values; from an average of 7.3 to 8.1 (Figure 3, columns C and D). Inclusion of these pre-excision steps significantly increased RIN values by reducing RNA degradation because these were applied prior to dissecting out the pancreatic tissue out of the body (Table 2 and Figure 5). Further, the excision step was also required to be carried out rapidly to reduce RNA degradation. As we became more efficient with handling and isolating the pancreas, the quality of the extracted pancreatic RNA improved significantly over time.

While the procedure involving perfusion and clamping of the duodenum was similar to Mullin et al. [4], we added two amendments to the optimized RNA extraction protocol. The first was to use cold QIAzol to enhance its effectiveness and reduce the high levels of endogenous nuclease activity. The extracted tissue was also rinsed with RNA*later* (Qiagen) prior to placing it in ice-cold QIAzol. In the protocol developed by Mullin et al. [4], the volume of QIAzol had been increased to 5 mL and used in a ratio of approximately 30 mg tissue to 5 mL liquid prior to homogenization. Therefore, we increased the volume further to test if it would enhance the RIN value of the extracted RNA. We tested two volumes of QIAzol (5 and 7 mL) in two independent experiments using approximately the same weight of tissue (<50 mg). However, increasing the volume of QIAzol reduced the RIN value from 8.1 ± 0.1 to 7.2 ± 0.2 (Table 2, methods D and E). It is possible that the increase in QIAzol resulted in phenolic contamination of the sample, since phenol is one of the components of QIAzol. Increasing QIAzol volume would thus require additional washing steps to remove phenol. Therefore, to save time and cost, only 5 mL QIAzol was used in the optimized protocol thereafter.

The second modification was a centrifugation step performed after homogenizing the tissue with the lysis reagent, which was adapted from Azevedo-Pouly et al. [8] who reported that time could be saved if, instead of dissociating the entire tissue, the lysate is centrifuged to remove unhomogenized fragments, thus avoiding RNA degradation. Similarly, we included this centrifugation step to pellet out any undigested tissue, which might affect the quality of the extracted RNA as a modification of the RNA extraction method using the RNeasy Mini Kit (Qiagen) (Figure 2).

The concentration of RNA extracted from healthy and diabetic rats showed some variation in our improved protocol (Table 3 and Figure 6A,B). Although both groups (healthy and diabetic rats) had approximately similar RIN values (8.2 and 8.3, respectively), the amount of RNA extracted from healthy rats was higher than that extracted from diabetic rats (455.0 ± 83.2 and 295.1 ± 64 ng/µL, respectively). Cefalo et al. [10] presented similar results when comparing RNA extracted from frozen human pancreatic samples of non-diabetic and diabetic patients who had undergone partial pancreatectomy. By means of the advanced technology of pancreatic imaging, it was revealed that the pancreas of patients with type 1 or type 2 diabetes had an overall reduced volume as compared to that of healthy individuals [11], which might explain the difference in the amount of RNA observed in this study.

The cDNA synthesized from pancreatic RNA isolated using different extraction protocols was used in RT-PCR to amplify the housekeeping *β-actin* gene; the differences in its amplification reflected the effect of the modifications on the quality of the pancreatic RNA extracted. Following comparison with the control rat liver RNA that was extracted using the standard RNeasy Mini Kit (Qiagen) (Figure 7, lane 8) with that extracted using the various published protocols, it was clear that our modified RNA protocol yielded the best results (Figure 7, lanes 9–14), as compared to pancreatic RNA extracted using the standard protocol that resulted in RNA of very low integrity and quality (Figure 7, lanes 1–3). Moreover, injecting RNAlater (Qiagen) without clamping the HPD, and increasing the QIAzol volume, hardly improved the intensity RT-PCR products (Figure 7, lanes 4 and 5). Additionally, injecting RNAlater with clamping the HPD and increasing the QIAzol volume from 5 to 7 mL yielded inconsistent results with variable band intensities (Figure 7, lanes 6–7). The integrity of the RNA isolated from healthy and diabetic rats was similar, yielding consistent and reproducible RT-PCR results (Figure 7, lanes 9–14), reflecting the efficacy of our optimized pancreatic RNA extraction method under different pathological conditions. This method is thus valuable in studies related to diabetes, as it is reliable and solves several challenges related to pancreatic RNA extraction.

Following our protocol, we were able to approximately double the RIN value of the extracted pancreatic RNA using the RNeasy Mini Kit (Qiagen) without any modifications. The improved RNA extraction protocol is reproducible and effective despite the additional cost of the QIAzol step, which is necessary to inhibit the high level of endogenous pancreatic RNase activity. All modifications to the standard protocol performed in this study were compared to those reported in other studies from which some steps were adapted (Table 4).

## 5. Conclusions

Our optimized pancreatic RNA extraction protocol using the RNeasy Mini Kit (Qiagen) significantly increased RIN values as compared to using the TriPure isolation reagent (Roche) and the RNeasy Mini Kit (Qiagen) without modifications and is a reliable and reproducible technique for isolating pancreatic RNA of superior quality and integrity. This is supported by the analysis of the quality of extracted RNA as well as the amplicons generated using RT-PCR of cDNA synthesized from the extracted RNA. Pancreatic RNA of high quality is essential for complex downstream gene expression studies in healthy and diabetic rat models. RNA isolated in this study is suitable for use in microarray analysis and RNA-Seq by next-generation sequencing.

## Figures and Tables

**Table 1:** A summary of published protocols for pancreatic RNA extraction.

No.	Study	Source	Treatment with RNA*later*	Tissue Preservation	Extraction Method	RNA Yield	Absorbance Ratio A260/A280 nm	RNA Integrity Assessment
1	Augereau et al., 2016 [3]	Mice	A quarter of the pancreas was removed and injected with 500 µL RNA*later*.	Small pieces of the injected pancreas were placed in 350 µL RNA*later*, snap-frozen in liquid nitrogen, and stored at −80 °C for 24 h.	Guanidium thiocyanate-phenol extraction using TriPure isolation reagent.	10–15 mg	1.85 ± 0.01	RIN = 8.9 ± 0.38 using the Agilent 2100 bioanalyzer system.
2	Mullin et al., 2006 [4]	Mice	1–2 mL RNA*later* was injected through the common bile duct after clamping the duodenum at the sphincter of Oddi.	The excised part of the pancreas (30 mg) was placed in 5 volumes of RNA*later* on ice and processed immediately for extraction.	Guanidium thiocyanate-phenol extraction using TRIzol reagent.	4.97 ± 1.92 µg/mL	1.41 ± 0.06	Clear bands of the ribosomal 28S and 18S RNA subunits were detected on 0.8% agarose gel.
3	Kiba et al., 2007 [5]	Rats	The pancreas was snap-frozen in liquid nitrogen and placed in 10 volumes of RNA*later*-ICE.	The tissue was processed either immediately, 30 min later, after overnight at 4 °C, or following storage at −80 °C for 1–7 d.	Qiagen RNeasy Mini Kit	0.5–1 µg/mL	-	Clear bands of the ribosomal 28S and 18S RNA subunits were determined by laser densitometry.
4	Griffin et al., 2012 [6]	Mice and piglets	Half the pancreas was excised and perfused with RNA*later* at multiple sites.	The perfused pancreas was cut into small pieces, snap-frozen in liquid nitrogen, and stored at −80 °C.	Qiagen RNeasy lipid tissue Mini Kit	Mice: 697 ± 203 ng/µL Piglets: 115 ± 17 ng/µL	-	Average RIN: mice around 6.5 piglets around 8
5	Dastgheib et al., 2014 [7]	Rats	The pancreas was immersed in 1 mL RNAl*ater* and cut into small pieces (20–30 mg).	The tissue was processed immediately, 30 min later, after overnight at 4 °C, or following storage at −80 °C for 1–7 days.	Several methods: RNX-plus solution, TriPure, and Qiagen RNeasy micro kits	-	-	Clear bands of the ribosomal 28S and 18S RNA subunits were detected on denaturing agarose gel from samples immersed in RNA*later* for 24 h at −80 °C and extracted by TriPure reagent; the cDNA quality was confirmed with (*β-actin*) amplification by RT-PCR.
6	Azevedo-Pouly et al., 2014 [8]	Mice	This study did not use RNA*later* for improving RNA integrity but, rather, recommendations and modification steps in the extraction from the RNeasy Mini Kit that were used in our optimized extraction protocol.			20–40 µg	Approx. 2.0	RIN = 7.4 ± 0.20 using the Agilent 2100 Bioanalyzer System and confirmed with qRT-PCR for three housekeeping genes.

RNA*later* (Qiagen, Hilden, Germany); TriPure isolation reagent (Roche, Basel, Switzerland).

**Table 2:** Quantification of RNA from rat pancreas following different excision techniques and extraction protocols.

RNA Extraction Method	Modification(s)	Sample Source	RIN	Conc. ng/µL	Absorbance Ratio A260/A280	Absorbance Ratio A260/A230
RNeasy Mini Kit	-	NR	3.6 ± 0.8	812.7 ± 30.1	1.95 ± 0.08	1.49 ± 0.24
TriPure	RNA*later* pancreatic injection after excision	NR	3.7 ± 0.8	1263.0 ± 387.4	1.78 ± 0.14	1.00 ± 0.13
RNeasy Mini Kit	RNA*later* injection into BD without clamping + homogenization with 5 mL QIAzol	NR	7.3 ± 0.5	1640.7 ± 436.3	2.12 ± 0.01	2.01 ± 0.07
RNeasy Mini Kit	RNA*later* injection into BD with clamping + homogenization with 5 mL QIAzol + centrifugation after tissue homogenization	NR	8.1 ± 0.1	391.0 ± 55.9	2.09 ± 0.01	1.96 ± 0.05
RNeasy Mini Kit	RNA*later* injection into BD with clamping + homogenization with 7 mL QIAzol + centrifugation after tissue homogenization	NR	7.2 ± 0.2	595.7 ± 164.9	2.11 ± 0.01	1.91 ± 0.08

Results are reported as mean ± SEM. BD, bile duct. RIN, RNA integrity number; NR, normal healthy rats; TriPure isolation reagent (Roche, Basel, Switzerland).

**Table 3:** Analysis of RNA extracted from healthy or diabetic rats.

Sample	Number	Concentration ng/µL	RIN	Absorbance Ratio A260/A280 nm	Absorbance Ratio A260/A230 nm
NR	6	455.0 ± 83.2	8.2 ± 0.05	2.09 ± 0.01	1.86 ± 0.08
DR	6	295.1 ± 64.8	8.3 ± 0.17	2.07 ± 0.01	1.86 ± 0.05

NR, normal healthy rats; DR, diabetic rats.

**Table 4:** Comparison of the experimental steps in the current study with that in two other studies from which the steps were adapted.

Experimental Steps	Current Protocol	Mullin et al., 2006 [4]	Azevedo-Pouly et al., 2014 [8]
Pancreas source	Rats	Mice	Mice
Clamping of the HPD	✓	✓	-
RNAlater injection	Pre-excision	Pre-excision	-
Weight of pancreas	<50 mg	30 mg	Entire pancreas was excised
Rinsing with RNAlater after excision	✓	-	-
Volume of lysis buffer	5 mL ice-cold QIAzol	5 mL TRIzol	8 mL ice-cold QIAzol
Extra centrifugation step after homogenization with lysis buffer	✓	-	✓
RIN value	8.2 ± 0.05	Not specified	7.4 ± 0.20

HPD, hepatopancreatic duct; RNA*later* (Qiagen, Hilden, Germany); QIAzol (Qiagen, Hilden, Germany).

## Data Availability

The authors declare that all data supporting the findings of this study are available within the article or from the corresponding author upon reasonable request.
